# Wahrnehmungen zum Umgang mit Opioiden: Fokus COVID-19: Freitext-Analyse einer Umfrage in Anästhesiologie/Intensivmedizin, Innerer Medizin und Palliativmedizin

**DOI:** 10.1007/s00482-021-00620-y

**Published:** 2022-01-24

**Authors:** Vera Peuckmann-Post, Carolin Hagedorn, Norbert Krumm, Roman Rolke, Frank Elsner

**Affiliations:** 1 Klinik für Palliativmedizin, Medizinische Fakultät, RWTH Aachen University, Aachen, Deutschland; 2 Klinik für Anästhesiologie, Medizinische Fakultät, RWTH Aachen University, Aachen, Deutschland; 3 Klinik und Poliklinik für Frauenheilkunde, Universitätsklinikum Leipzig, Leipzig, Deutschland

**Keywords:** Dyspnoe, Symptomkontrolle, Qualitative Analyse, Palliativmedizin, Survey, Dyspnea, Symptom management, Qualitative analysis, Palliative care, Survey

## Abstract

**Hintergrund:**

Obwohl Opioide wirksam Schmerzen und Dyspnoe lindern, findet dies in Leitlinien zur Symptomkontrolle unterschiedliche Gewichtung. Hierdurch können auch bei COVID-19 Unsicherheiten bezüglich Indikationen und ethischer Implikationen im Umgang mit Opioiden entstehen.

**Ziel der Arbeit:**

Wir untersuchten die persönliche Wahrnehmung des Umgangs mit Morphin/Opioiden (M/O) zur Symptomkontrolle *inner- und außerhalb* der Palliativmedizin (PM), auch bei der Betreuung COVID-19-Erkrankter, durch Befragung von Mitgliedern der Fachgesellschaften für Palliativmedizin, Innere Medizin, Anästhesiologie und Intensivmedizin.

**Material und Methoden:**

Mittels Survey Monkey® (Momentive Inc., San Mateo, CA, USA) wurden die Mitglieder anonymisiert nach ihrer eigenen Wahrnehmung des Umgangs mit M/O zur Symptomkontrolle befragt. Diese Ergebnisse wurden bereits publiziert. Zur systematischen und strukturierten Auswertung aller Kommentare des Freitextfelds wurde Phillip Mayrings Methode der zusammenfassenden qualitativen Inhaltsanalyse gewählt.

**Ergebnisse und Diskussion:**

Von 2202 Personen schrieben 339 im Freitextfeld „Weitere Anmerkungen“ zusätzliche Kommentare. Das induktiv entwickelte Kategoriensystem umfasste fünf Hauptkategorien: 1) Eigene Wahrnehmungen mit COVID-19-Erkrankten, 2) Anwendungsgebiet und Wirkung von M/O, 3) Beobachtungen im Bereich der Palliativmedizin, 4) Vermittlung von Wissen zum Umgang mit Opioiden bzw. Palliativmedizin, und 5) Ergänzendes.

**Fazit:**

Einige Teilnehmende berichteten sehr persönliche Wahrnehmungen und wiesen insbesondere im Umgang mit COVID-19 auf Defizite im Gesundheitssystem hin. Einheitliche interdisziplinäre Leitlinien zur Symptomkontrolle, mehr Lehre und Unterstützung durch in der Symptomkontrolle kompetente Expert:innen erscheinen erforderlich.

## Abstract (en)

**Background:**

Opioids efficiently manage pain and dyspnea. However, guidelines on symptom management with opioids differ, which may lead to uncertainty among medical staff concerning opioid indication and ethical implication, especially when caring for COVID-19 patients.

**Aims:**

We aimed to examine the perception of morphine/opioid (M/O) administration for symptom control *within* and *outside* palliative care, including care for COVID-19 patients, among members of the German associations for palliative medicine, internal medicine, anesthesiology and intensive care.

**Methods:**

Participants received an anonymized online questionnaire via Survey Monkey® (Momentive Inc., San Mateo, CA, USA) regarding their general perception of symptom management with M/O. These results have been published elsewhere. For systematic and structural analysis of comments in the free-text field, we chose Phillip Mayringʼs method of summarizing qualitative content analysis.

**Results:**

Of the* n* = 2202 persons who participated, 339 wrote comments in the free-text field which were categorized as follows: main categories 1) personal perceptions of COVID-19 patients, 2) administration and effect of M/O, 3) observations within the palliative care field, 4) imparting knowledge concerning M/O usage and palliative care, and 5) others.

**Conclusions:**

Some participants reported very personal perceptions and deficits of the healthcare system, especially when caring for COVID-19 patients. Uniform interdisciplinary guidelines for symptom control, more education, and support by trained staff confident in symptom control should be increasingly considered in the future.

## Einleitung

Eine sichere Anwendung von Opioiden zur Symptomkontrolle ist wichtig, denn sie lindern wirksam Schmerzen und Dyspnoe [2, 12, 15, 18, 19]. Hingegen können sich Unsicherheiten in der Opioidanwendung sowie Unkenntnis von Leitlinien in einer heterogenen Patientenversorgung abbilden [7]. Bei der COVID-19(Coronavirus SARS-CoV‑2)-Erkrankung ist die sichere Kenntnis zur Therapie der Dyspnoe besonders bedeutsam [1, 19]. Daher führten wir zum Beginn der zweiten Pandemiewelle eine Umfrage zur Wahrnehmung des Umgangs mit Morphin/Opioiden (M/O) unter Mitgliedern der Fachgesellschaften für Palliativmedizin, Innere Medizin, Anästhesiologie und Intensivmedizin durch [23, 24]. Die quantitativen Daten bestätigten eine teils große Unsicherheit, welche wir nun durch qualitative Analyse der persönlichen Kommentare im Freitextfeld „Weitere Anmerkungen“ zur Umfrage näher charakterisierten.

## Methode

Auf Grundlage von Interviews mit Ärzt:innen und Pflegenden innerhalb wie außerhalb der Palliativmedizin (PM) (Einzelinterviews und Fokusgruppeninterview, nicht publiziert) sowie auf der Grundlage internationaler Literatur identifizierten wir häufige Fragestellungen und Assoziationen zur Wahrnehmung des Umgangs mit Morphin/Opioiden (M/O) in der Symptomkontrolle. Aus diesen entwickelten wir einen elektronischen Fragebogen, und sechs Personen (vier Ärzt:innen, zwei Pflegende) testeten die Benutzerfreundlichkeit und technische Funktionalität vor dem Einsatz.

Um einen Vergleich innerhalb versus außerhalb der PM abbilden zu können, kontaktierten wir folgende Fachgesellschaften: Deutsche Gesellschaft für Palliativmedizin (DGP), Deutsche Gesellschaft für Anästhesiologie und Intensivmedizin (DGAI), Berufsverband Deutscher Anästhesisten e. V. (BDA) und Deutsche Gesellschaft für Innere Medizin (DGIM). Die Mitglieder erhielten eine E‑Mail über die jeweiligen E‑Mail-Verteiler der Sekretariate mit einem Link zu einer freiwilligen, anonymisierten Online-Umfrage (Survey Monkey®; Momentive Inc., San Mateo, CA, USA) in den Monaten September bis November 2020. Zur besseren Charakterisierung der Teilnehmenden baten wir das Ausmaß einer Tätigkeit in der PM („gar nicht“, „teilweise“, „vollständig/überwiegend“) anzugeben.

Im Fragebogen erläuterten wir Begriffe wie „innerhalb“/„außerhalb“ der PM, und informierten über Initiatoren, Zeitangabe zur Durchführung und Anonymisierung. Die Eingabe erfolgte manuell, und es war möglich, einzelne Fragen unbeantwortet zu überspringen sowie im Freitextfeld „Weitere Anmerkungen“ Kommentare zu hinterlassen. Die Teilnahme war nur einmalig möglich (IP-Adressen-Registrierung durch SurveyMonkey®).

Zur systematischen und strukturierten Auswertung aller Kommentare des Freitextfelds wurde Phillip Mayrings Methode der zusammenfassenden qualitativen Inhaltsanalyse gewählt [20]. Alle Inhalte wurden im Sinne eines induktiven Vorgehens in einem Kategoriensystem mit Hauptkategorien, Sub-Kategorien und Sub-Sub-Kategorien strukturiert. Die Analyse erfolgte anschließend manuell und das Anmerkungsmaterial wurde in zwei Durchgängen analysiert. Geprüft wurden das Kategoriensystem, die inhaltliche Strukturierung und Codierungen nachfolgend durch eine zweite Autorin. Bei Dissens stellte die zweite Autorin das Thema zur Diskussion im Autor:innen-Team, bis ein Konsens gefunden wurde.

## Ergebnisse

Analysiert wurden insgesamt 339 Kommentare von 2202 Teilnehmenden. Bei DGP-Teilnehmenden handelte es sich primär um Ärzt:innen und Pflegende, während bei DGAI/BDA und DGIM primär Ärzt:innen teilnahmen. In der Tab. 1 sind 46 Ankerbeispiele (Zitate als Beispiel für die jeweilige Kategorie) aufgeführt, die von Ärzt:innen, Pflegenden sowie zwei weiteren Personen berichtet wurden.

**Table 1:** 

Hauptkategorie	Subkategorie	Sub-Sub-Kategorie	Ankerbeispiel
*(1) Eigene Wahrnehmungen mit COVID-19-Erkrankten*	Kein Kontakt zu COVID-19-Erkrankten	–	Keine Erfahrung mit COVID-19-Erkrankten. (Nr. 45/723, DGAI: Arzt, Krankenhaus, Intensivmedizin, Nicht-PM)
	Keine Erfahrung mit COVID-19 und Palliativmedizin	–	Auf unserer Palliativeinheit wurden keine COVID-Patienten betreut. […] (91/113, DGP: Ärztin, Krankenhaus, vollständig/überwiegend PM)
	Persönliche Erfahrungen mit COVID-19-Erkrankten	Verlegung auf andere Stationen	COVID-19-Erkrankten-bezogene Fragen können nicht beantwortet werden, da bei positiven Befunden die Patienten in eine andere Klinik verlegt werden (13/230, DGIM: Ärztin, Krankenhaus, teilweise PM-Bereich)
		Intensivmedizinische Betreuung	Zum Einsatz von Morphin bei COVID-19-Erkrankten in der Palliativmedizin kann ich keine Aussagen machen, da ich diese Patienten nur intensivmedizinisch und notfallmedizinisch versorgt habe. (52/1.005, DGAI: Arzt, Anästhesie/OP, Intensivmedizin, Notfallmedizin, teilweise PM-Bereich)
		Verlängerung des Sterbeprozesses	Vor allem in Zusammenhang mit COVID-19 wird der Sterbeprozess bei infausten Prognosen und Patienten mit Mehrorganversagen prolongiert, da man durch die initiale Unklarheit der Pathophysiologie und genereller Schwere der Erkrankung alles bis zum Letzten ausgereizt hat (Beatmung, Tracheotomie, CVVHDF, antibiotische Therapie, Katecholamin-Therapie, etc.) bis man letztlich sich doch eingestehen musste, dass man bei wenigen Leuten den Kampf doch verloren hat. Ohne die (Neben)Diagnose COVID-19-Pneumonie hätten wir den Menschen und auch ihren Angehörigen viel früher den Palliativen Weg bei mehrwöchigem frustranen Therapieversuchen gebahnt. (146/352, DGAI: Ärztin, Krankenhaus, Intensivmedizin, teilweise PM-Bereich)
		Symptomorientierte Therapie	Ich denke [COVID-19] Patienten werden anhand ihrer Symptome behandelt, auch was Schmerztherapie angeht. (106/632, DGAI: Ärztin, Krankenhaus, Anästhesie/OP, Schmerztherapie, Nicht-PM-Bereich)
		Vergleichbare Therapie mit Patient:innen mit pulmonalen Erkrankungen	Bei uns werden COVID-19-Patienten nicht anders behandelt als andere Patienten mit Lungenerkrankungen. (170/120, DGAI: Ärztin, Krankenhaus, Anästhesie/OP, Intensivmedizin, teilweise PM-Bereich)
	Erfahrungen mit COVID-19 und M/O- Anwendung	Keine Erfahrungen mit COVID-19 und M/O	Ich habe keinerlei Erfahrung im Einsatz von Morphin bei COVID-19 Erkrankten. (5/297, DGIM: Ärztin, Praxis, teilweise PM-Bereich)
		–	„Wir haben einige Dutzend COVID-19-Patienten betreut und hatten eher den Eindruck, dass das subjektive Gefühl der Luftnot im Vergleich zum klinischen Eindruck (Tachypnoe, Einsatz der Atemhilfsmuskulatur, erniedrigter pCO_2_ in der BGA) oft ungewöhnlich gering ausgeprägt ist, deshalb waren wir bisher auch eher unsicher/zurückhaltend mit dem Einsatz von Opiaten außerhalb der Palliativsituation oder Intensivstation, da natürlich nicht.“ (44/342, DGP: Ärztin, Krankenhaus, teilweise PM-Bereich)
		M/O als wichtiger Bestandteil bei COVID-19-Erkrankten	Ein Verzicht auf Morphin in der COVID-19-Therapie ist meines Erachtens fahrlässig, […] (88/125, DGP: weibliche Pflegekraft, Altenpflegeeinrichtung, teilweise PM-Bereich)
*(2) Anwendungsgebiet und Wirkung von M/O*	Kein Unterschied der Anwendung von M/O bei COVID-19-und Nicht-COVID-19-Erkrankten	–	Ich wüsste nicht, warum der Einsatz von Morphin [bei COVID-Erkrankten] anders sein sollte als bei Patienten mit metastasiertem Lungenkarzinom, Lungenfibrose oder schwerer COPD. (25/144, DGIM: Ärztin, SAPV, AAPV, Onkologie, teilweise PM-Bereich)
	Symptomkontrolle	Dyspnoe	Morphin bevorzuge ich in der Palliativmedizin gegenüber anderen Opioiden wegen der guten Wirkung, um Dyspnoe und Angstzustände zu behandeln. (28/1.159, DGAI: Arzt, Krankenhaus, Anästhesie/OP, teilweise PM-Bereich)
		Angstzustände	[Morphin] hilft wunderbar gegen die Atemnot und Angst […], (88/125, DGP: weibliche Pflegekraft, Altenpflegeeinrichtung, teilweise im PM-Bereich tätig)
		Beruhigung während des Sterbeprozesses	Meine Erfahrung z. B. über 30 Jahre mit Patienten in diversen grenzwertigen Lebenssituationen schafft Vertrauen und eine verlässliche Intuition im Einsatz von Morphin zum richtigen Zeitpunkt und in der Dosis für den Patienten, die ihm gut tut zum Leben oder ihm bei infauster Prognose das Sterben erleichtern kann. Im letzteren Fall zahlt dann würdiger Weise der ruhige, friedliche Ausdruck des Menschen und nicht die zu beobachtende Atemdepression, wie nicht selten von unerfahrenem Pflegepersonal im Pflegebericht leider dokumentiert wird. (37/1.109, DGAI: Ärztin, Krankenhaus, Anästhesie/OP, Intensivmedizin, Notfallmedizin, teilweise PM Bereich)
		Supportiver Bestandteil des Sterbens	Dabei ist Morphin immer nur Adjuvans für den indizierten bedürftigen Zustand eines Menschen, nie ein Macher von Leben oder Tod. (37/1.109, DGAI: Ärztin, Krankenhaus, Anästhesie/OP, Intensivmedizin, Notfallmedizin, teilweise PM-Bereich)
		Erhöhung Lebensqualität/Vermeidung des Leidens	Mut zur Therapie des Leidens (12/1.304, DGAI: Arzt, Krankenhaus, Anästhesie/OP, Intensivmedizin, Nicht-PM-Bereich)
		Atemdepression als therapeutische Stärke	Ein Analgetikum mit der Potenz von Opioiden wäre wünschenswert in manchen Bereichen, die Atemdepression ist beim sinnvollen Umgang mit Morphin eine therapeutische Stärke. (74/876, DGAI: Arzt, Krankenhaus, teilweise PM-Bereich)
	Angstbesetzte und zurückhaltende Anwendung	–	Der (korrekte) Umgang mit Opioiden ist außerhalb der Palliativmedizin und der Schmerztherapie leider meiner Erfahrung nach sehr unbeholfen. Wenn nicht zufällig ein Interesse oder eine gezielte Fortbildung auf diesem Bereich besteht, wissen die meisten ärztlichen Kollegen wenig über Anwendung, Dosierung, Wechselwirkung und insbesondere Nutzung von Nebeneffekten für therapeutische Zwecke. Das ist sehr schade und sollte sich ändern. […] (97/709, DGAI: Arzt, Krankenhaus, Anästhesie/OP, Intensivmedizin, Palliativstation, teilweise PM-Bereich)
	–	–	[…] Wenn Morphin mutiger bei COVID-19 Patienten eingesetzt worden wäre, hätte stationär so manche Beatmung verhindert werden können! […] (Nr. 88/125, DGP: weibliche Pflegekraft, teilweise im PM-Bereich tätig, Altenpflegeeinrichtung)
	Unsicherheiten	–	Außerhalb des Palliativbereiches leider oft aus Unsicherheit Verzögerung oder Verzicht auf Morphin. (Nr. 14/228, DGIM: Ärztin, Krankenhaus, teilweise PM-Bereich)
	Wissensmangel und Interessensmangel	–	Lehre bedeutet Schulen und Lernwilligkeit, besonders bei Kollegen trifft man immer wieder auf desinteressierte Besserwisserei. (8/1.321, DGAI: Ärztin, Krankenhaus, teilweise PM-Bereich)
	–	–	Es wäre sehr wünschenswert, wenn alle Kollegen und Kolleginnen sich verpflichtet fühlen würden, ein Grundwissen über den Einsatz von Morphin zu pflegen und in der Lage wären, BTM-Rezepte auszustellen. […] (110/599, DGAI: Ärztin, Praxis, SAPV, AAPV, anderes ambulantes Team, Hospiz, Altenpflegeeinrichtung, Ethikkommission, teilweise PM-Bereich)
	Notwendigkeit adäquater Analgesie	–	Jedes Haus sollte eine Schmerzambulanz besitzen, die bei etwaigen Problemen zur Hilfe bereitsteht und chronische Schmerzpatienten mitbetreut, am ehesten aus dem anästhesiologischen Bereich. (58/971, DGAI: Arzt, Krankenhaus, Anästhesie/OP, Intensivmedizin, Schmerzambulanz, Nicht-PM-Bereich)
	Sterbebeschleunigung durch M/O	M/O und beschleunigter Sterbeprozess	Ich sehe keine Indikation für Morphin, das Sterben zu beschleunigen […] (9/1.319, DGAI: Arzt, Krankenhaus, Anästhesie/OP, teilweise PM-Bereich)
		M/O zur Symptomkontrolle, beschleunigt den Sterbeprozess nicht	[…] [Morphin] lindert die Symptome, erleichtert, beschleunigt aber den Sterbeprozess nicht. (13/230, DGIM: Ärztin, Krankenhaus, teilweise PM-Bereich)
	Fehlgebrauch/Missbrauch von Opioiden	–	Ich wünschte mir mehr Kontrolle in beispielsweise Pflegeheimen […] von „Fachkräften“, die sich angeblich auskennen – sehr problematisch! (38/365, DGP: Dozentin für Pflegeberufe, Fachpflegeschule, Nicht-PM-Bereich)
			Ich wünsche mir ein Werbeverbot für Opioide. (143/386, DGAI: Arzt, Schmerzambulanz, teilweise PM-Bereich)
			Wir haben vor vermehrtem Einsatz gewarnt, um möglichst keine US-Verhältnisse in Deutschland zu bekommen! (48/1.041, DGAI: Arzt, Krankenhaus, Anästhesie/OP, Intensivmedizin, Schmerzambulanz, Rettungsmedizin, teilweise PM-Bereich)
			Sorge um Missbrauch ernst nehmen, Ängste abbauen. (42/353, DGP: männliche Pflegekraft, Krankenhaus, teilweise PM-Bereich)
*(3) Beobachtungen im Bereich der Palliativmedizin*	Palliativteams/Konsildienste	–	In unserer Einrichtung sind die Bedingungen sehr gut und werden im Bedarfsfall sogar optimiert, erfahrene Palliativfachkräfte unterstützen und koordinieren jeden absehbaren Sterbeprozess. Sie begleiten die Pflege- und Betreuungsteams. (3/483 DGP: weibliche Pflegekraft, Altenpflegeeinrichtung, teilweise PM-Bereich)
	Teamarbeit	–	Teamkonferenzen etc. sind sehr wichtig! Da ich allerdings auch AAPV und SAPV mache, habe ich aufgrund der Organisationsstruktur für mich ausreichende Supervisionen, Teamsitzungen und bin selbst Team des Palliativkonsildienstes. (33/383, DGP: Ärztin, Praxis, teilweise PM-Bereich)
	Interdisziplinäres Arbeiten	–	Bessere Zusammenarbeit zwischen ITS [Intensivstation] und Palliativ, mehr Teilnahme vor allem von Ärzten an palliativmedizinischen Fortbildungen (92/92, DGP: Ärztin, Palliativzentrum, vollständig/überwiegend PM-Bereich)
	Zu späte Einbeziehung der Palliativmedizin	–	Ein palliativmedizinischer Ansatz wird nach meiner Einschätzung in der Klinik zu spät gemacht und umgesetzt (Langjährige Anästhesistin/Intensivmedizinerin) (81/1.384, DGAI: Ärztin, Praxis/AAPV, teilweise PM-Bereich)
	Ausbaufähige palliativmedizinische Versorgung	Notwendige flächendeckende Versorgung	Es hapert an allen Enden. Die palliativmedizinische Versorgung ist nach wie vor flächendeckend unzureichend. Gerade die Hausärzte versuchen immer wieder, die [Morphin-]Entscheidung auf den Notarzt abzuwälzen. (111/585, DGAI: Arzt, Krankenhaus, Praxis, Anästhesie/OP, Intensivmedizin, Schmerzambulanz, Notfallmedizin, teilweise PM-Bereich)
	Notwendigkeit von Ausbildung und einheitliche Vermittlung von Empfehlungen	Versorgung in Kliniken	Auf der politischen und Krankenhausleitungsebene sollte an mehr palliativmedizinischen Betten und palliativmedizinischen Personal (Ärzte und Pflege) nicht nur gedacht, sondern auch in die Tat umgesetzt werden. (140/404, DGAI: Arzt, Krankenhaus, Anästhesie/OP, Intensivmedizin, teilweise PM-Bereich)
*(4) Vermittlung von Wissen zum Umgang mit M/O bzw. Palliativmedizin*	Adressaten für Wissen	Medizinisches Personal (Pflege, Ärzt:innen, Psycholog:innen etc.)	Breitflächigere Informationen aller beteiligten Fachgruppen von der Pflegehelferin bis zum Chefarzt aller Abteilungen, da oft Unwissenheit herrscht. (107/626, DGAI: Ärztin, Krankenhaus, Schmerztherapie, Notfallmedizin, teilweise PM-Bereich)
	–	Bereiche außerhalb der Palliativmedizin	Der (korrekte) Umgang mit Opioiden ist außerhalb der Palliativmedizin und der Schmerztherapie leider meiner Erfahrung nach sehr unbeholfen. […]. Mehr Ausbildung wäre hier unbedingt angezeigt. (97/709, DGAI: Arzt, Krankenhaus, Anästhesie/OP, Intensivmedizin, Palliativstation, teilweise PM-Bereich)
	–	Patient:innen	Im Telemonitoringbereich gibt es auch Patienten mit COPD oder Herzinsuffizienz, denen ich auf Grund meiner Palliativerfahrung immer wieder Palliativtherapie empfehle, manche haben davon noch nie etwas gehört, andere verbinden es mit Medizinischer Betreuung von Sterbenden. (105, DGP: Arzt, vollständig/überwiegend im PM-Bereich tätig)
	–	Angehörige	Mehr Aufklärung des medizinischen Personals, Patienten und Angehörigen. (101/679, DGAI: Arzt, Krankenhaus, Anästhesie/OP, teilweise PM-Bereich)
	–	Allgemeinbevölkerung	Transparenz in der Bevölkerung und in der Politik, welche Berufsgruppen sich intensiv mit der Thematik auseinandersetzen und welche Instrumente zur Verbesserung benötigt werden. (Nr. 22, DGP: Arzt, teilweise PM-Bereich)
	Gewünschte Inhalte der Weiterbildung und Handlungsempfehlungen	Symptomkontrolle mit M/O	Lehre, sowohl über den Umgang mit Opioiden als auch über Palliativmedizin, sollte für sämtliche Ärzte Pflicht sein. (15/1.275, DGAI: Ärztin, Krankenhaus, Anästhesie/OP, Intensivmedizin, Notfallmedizin, teilweise PM-Bereich)
		Handlungsempfehlungen zu M/O im Sterbeprozess	Eine Handlungsempfehlung im Umgang mit Opioiden (im klinischen und ambulanten Setting) im Sterbeprozess fände ich sehr sinnvoll. (139/407, DGAI: Arzt, Krankenhaus, Anästhesie/OP, Intensivmedizin, Nicht-PM-Bereich)
		Leitlinien zu COVID-19 und Palliativmedizin	Sehr interessantes Thema. Würde Leitlinien bei COVID-19 und Palliativmedizin anschauen. (145/360, DGAI: Ärztin, Anästhesie/OP, Intensivmedizin, teilweise PM-Bereich)
		Konsens der Fachgesellschaften	Mehr Konsens bei den Fachgesellschaften wäre hilfreich! Es gab in den letzten Jahren in der Fachpresse eine 180-Grad-Wendung von „wir verordnen zu wenig Opioide“ zu „wir verordnen viel zu viele Opioide“, ohne dass für mich nachvollziehbar wurde, warum – und ohne klare Maßstäbe, was „zu viel“ oder „zu wenig“ ist (20/194, DGIM: Ärztin, Praxis, teilweise PM-Bereich)
*(5) Ergänzendes*	Eigener Eindruck	–	Juristisch eine bessere Absicherung in der heutigen Gesellschaft, die Klagefreudigkeit schafft doch die vielen Probleme, weshalb Mediziner verunsichert sind. (131/446, DGAI: Arzt, Krankenhaus, Praxis, Anästhesie/OP, Intensivmedizin, teilweise PM-Bereich)
	Themenvorschläge	–	Neue Themen, wie Morphin und Sport. (Nr. 23/1.199, DGAI: Ärztin, Krankenhaus, Anästhesie/OP, teilweise PM-Bereich)

### Kategoriensystem

Das induktiv entwickelte Kategoriensystem umfasste fünf Hauptkategorien:

1. Eigene Wahrnehmungen mit COVID-19-Erkrankten
2. Anwendungsgebiet und Wirkung von M/O
3. Beobachtungen im Bereich der Palliativmedizin
4. Vermittlung von Wissen zum Umgang mit M/O bzw. Palliativmedizin
5. Ergänzendes

Diese 5 Hauptkategorien beinhalteten insgesamt 22 Sub-Kategorien und 26 Sub-Sub-Kategorien, wobei beachtet werden muss, dass nicht jede Sub-Kategorie in Sub-Sub-Kategorien untergliedert wurde. Sub-Kategorien sind im Folgenden **fett** markiert, Sub-Sub-Kategorien *kursiv* geschrieben und die Gesamtergebnisse in Tab. 1 dargestellt. Im Text aufgeführte Beispielzitate wurden mit der Kommentarnummer und Zugehörigkeit zur Fachgesellschaft aufgelistet und bezüglich Rechtschreibung und sprachlicher Fehler korrigiert.

#### (1) Eigene Wahrnehmungen mit COVID-19-Erkrankten

Ein Teil der Teilnehmenden kommentierte, dass bisher **kein Kontakt zu COVID-19-Erkrankten** bestanden habe bzw. sie **keine Erfahrungen mit COVID-19 und der Palliativmedizin** gehabt hätten. **Persönliche Erfahrungen mit COVID-19-Erkrankten** beinhalteten Beschreibungen, in denen COVID-19-Erkrankte häufig bei positivem Testergebnis *auf eine andere Station verlegt *oder *intensivmedizinisch betreut* wurden. Ein Kommentar beschrieb, dass die COVID-19-Erkrankung zur *Verlängerung des Sterbeprozesses* geführt habe.

Vor allem in Zusammenhang mit COVID-19 wird der Sterbeprozess bei infausten Prognosen und Patienten mit Mehrorganversagen prolongiert, da man durch die initiale Unklarheit der Pathophysiologie und genereller Schwere der Erkrankung alles bis zum letzten ausgereizt hat (Beatmung, Tracheotomie, CVVHDF [Kontinuierliche veno-venöse Hämodiafiltration], antibiotische Therapie, Katecholamin-Therapie, etc.), bis man sich letztlich doch eingestehen musste, dass man bei wenigen Leuten den Kampf doch verloren hat. Ohne die (Neben‑)Diagnose COVID-19-Pneumonie hätten wir den Menschen und auch ihren Angehörigen viel früher den palliativen Weg bei mehrwöchigen frustranen Therapieversuchen gebahnt. (146/352, DGAI, Ärztin, Krankenhaus, Intensivmedizin, teilweise PM-Bereich)

Einige Teilnehmende beschrieben eine regelhafte *symptomorientierte Therapie* bei COVID-19-Erkrankten oder gingen darauf ein, dass Therapiekonzepte bei COVID-19-Erkrankten *vergleichbar mit Therapien bei Patient:innen mit pulmonalen Erkrankungen* seien.

Bei uns werden COVID-19-Patienten nicht anders behandelt als andere Patienten mit Lungenerkrankungen. (170/120, DGAI: Ärztin, Krankenhaus, Anästhesie/OP, Intensivmedizin, teilweise PM-Bereich)

Einzelne Teilnehmende beschrieben, dass sie *keine ***Erfahrung mit COVID-19 und der Anwendung von M/O** hätten. Andere betonten die *Wichtigkeit von M/O bei COVID-19-Erkrankten*.

Ein Verzicht auf Morphin in der COVID-19-Therapie ist meines Erachtens fahrlässig, es hilft wunderbar gegen die Atemnot und Angst und sollte auch bei leichteren Verläufen, die ambulant betreut werden, zum Einsatz kommen. Wenn Morphin mutiger bei COVID-19-Patienten eingesetzt worden wäre, hätte stationär so manche Beatmung verhindert werden können! […]. (88/125, DGP: weibliche Pflegekraft, Altenpflegeeinrichtung, teilweise PM-Bereich)

Wir haben einige Dutzend COVID-19 Patienten betreut und hatten eher den Eindruck, dass das subjektive Gefühl der Luftnot im Vergleich zum klinischen Eindruck (Tachypnoe, Einsatz der Atemhilfsmuskulatur, erniedrigter pCO_2_ in der BGA [Anmerkung: Blutgasanalyse] …) oft ungewöhnlich gering ausgeprägt ist, deshalb waren wir bisher auch eher unsicher/zurückhaltend mit dem Einsatz von Opiaten außerhalb der Palliativsituation oder Intensivstation, da natürlich nicht. (44/342, DGP: Ärztin, Krankenhaus, teilweise PM-Bereich)

#### (2) Anwendungsgebiet und Wirkung von M/O

Mehrere Kommentare gingen darauf ein, dass in der Anwendung von M/O **nicht zwischen COVID-19- und Nicht-COVID-19-Erkrankten unterschieden** werde.

Ich wüsste nicht, warum der Einsatz von Morphin [bei COVID-19-Erkrankten] anders sein sollte als bei Patienten mit metastasiertem Lungenkarzinom, Lungenfibrose oder schwerer COPD [Anmerkung: chronisch obstruktive Lungenerkrankung]. (25/144, DGIM: Ärztin, SAPV, AAPV, Onkologie, teilweise PM-Bereich)

Einige Teilnehmende sprachen über die Hauptindikation zur M/O-Gabe – die **Symptomkontrolle** – und spezifizierten dies mit Indikationen wie *Dyspnoe, Angstzuständen, zur Beruhigung während des Sterbeprozesses* und als *supportiven Bestandteil während des Sterbens* sowie zur *Erhöhung der Lebensqualität* bzw. *Vermeidung des Leidens*. Auch die Wirkung der *Atemdepression als therapeutische Stärke* wurde thematisiert.

Berichtet wurde ebenfalls von der Erfahrung, dass der Umgang mit M/O (außerhalb der Palliativmedizin/Schmerzmedizin) häufig **angstbesetzt** sei und Opioide eher **zurückhaltende Anwendung** fänden. Oft beobachtet und beschrieben wurden **Unsicherheiten** im Umgang mit Opioiden.

Der (korrekte) Umgang mit Opioiden ist außerhalb der Palliativmedizin und der Schmerztherapie leider meiner Erfahrung nach sehr unbeholfen. Wenn nicht zufällig ein Interesse oder eine gezielte Fortbildung auf diesem Bereich besteht, wissen die meisten ärztlichen Kollegen wenig über Anwendung, Dosierung, Wechselwirkung und insbesondere Nutzung von Nebeneffekten für therapeutische Zwecke. Das ist sehr schade und sollte sich ändern. […]. (97/709, DGAI: Arzt, Krankenhaus, Anästhesie/OP, Intensivmedizin, Palliativstation, teilweise PM-Bereich)

Einzelne Teilnehmende kritisierten vorherrschenden **Wissens- und Interessensmangel** im Umgang mit M/O unter Kolleg:innen. Die **Notwendigkeit adäquater Analgesie **wurde erläutert.

Es wäre sehr wünschenswert, wenn alle Kollegen und Kolleginnen sich verpflichtet fühlen würden, ein Grundwissen über den Einsatz von Morphin zu pflegen, und in der Lage zu wären, BTM-Rezepte auszustellen. […]. (110/599, DGAI: Ärztin, Praxis, SAPV, AAPV, anderes ambulantes Team, Hospiz, Altenpflegeeinrichtung, Ethikkommission, teilweise PM-Bereich)

Anmerkungen beinhalteten ebenfalls das Themenfeld **Sterbebeschleunigung durch M/O**: Manche Teilnehmenden waren sich einig, dass M/O den Sterbeprozess nicht beschleunige(n), andere sahen eine Sterbebeschleunigung durch M/O.

[…] [Morphin] lindert die Symptome, erleichtert, beschleunigt aber den Sterbeprozess nicht. […]. (13/230, DGIM: Ärztin, Krankenhaus, teilweise PM-Bereich)

Ich sehe keine Indikation für Morphin, das Sterben zu beschleunigen […]. (9/1.319, DGAI: Arzt, Krankenhaus, Anästhesie/OP, teilweise PM-Bereich)

Wenige Kommentare implizierten den **Fehlgebrauch** oder **Missbrauch von Opioiden:**

Ich wünsche mir ein Werbeverbot für Opioide. (143/386, DGAI: Arzt, Schmerzambulanz, teilweise PM Bereich)

Wir haben vor vermehrtem Einsatz gewarnt, um möglichst keine US-Verhältnisse in Deutschland zu bekommen! (48/1.041, DGAI: Arzt, Krankenhaus, Anästhesie/OP, Intensivmedizin, Schmerzambulanz, Rettungsmedizin, teilweise PM-Bereich)

Dagegen wünschten andere sich bezüglich dieses Aspekts einen weniger angstbehafteten Umgang mit Opioiden:

Sorge um Missbrauch ernst nehmen, Ängste abbauen. (42/353, DGP: männliche Pflegekraft, Krankenhaus, teilweise PM-Bereich)

Es wurden zudem juristisch problematische Zustände beschrieben:

Ich wünschte mir mehr Kontrolle in beispielsweise Pflegeheimen, […] von „Fachkräften“, die sich angeblich auskennen – sehr problematisch! (38/365, DGP: Dozentin für Pflegeberufe, Fachpflegeschule, Nicht-PM-Bereich)

#### (3) Beobachtungen im Bereich der Palliativmedizin

Kommentare bezogen sich immer wieder auf die Wichtigkeit von **palliativmedizinischen Konsilteams** an Kliniken und die **interdisziplinäre Arbeit** in der Betreuung von Patient:innen. Auch kommentierten einige gut funktionierende Supervisionen, **Teamarbeit **und Austausch untereinander.

Eine frühe Miteinbeziehung palliativmedizinischer Konzepte wurde von verschiedenen Teilnehmenden befürwortet und gleichzeitig beschrieben, dass die **Palliativmedizin teils zu spät in Behandlungen miteinbezogen** wurde.

Ein palliativmedizinischer Ansatz wird nach meiner Einschätzung in der Klinik zu spät gemacht und umgesetzt (Langjährige Anästhesistin/Intensivmedizinerin). (81/1.384, DGAI: Ärztin, Praxis/AAPV, teilweise PM-Bereich)

Teilnehmende beschrieben zudem **palliativmedizinische Angebote als ausbaufähig**, sowohl bezogen auf das *flächendeckende Angebot* als auch den Wunsch nach mehr palliativmedizinischen Betten und Personal *in Kliniken*.

Es hapert an allen Enden. Die palliativmedizinische Versorgung ist nach wie vor flächendeckend unzureichend. Gerade die Hausärzte versuchen immer wieder, die [Morphin-] Entscheidung auf den Notarzt abzuwälzen. (111/585, DGAI: Arzt, Krankenhaus, Praxis, Anästhesie/OP, Intensivmedizin, Schmerzambulanz, Notfallmedizin, teilweise PM-Bereich)

#### (4) Vermittlung von Wissen zum Umgang mit M/O bzw. Palliativmedizin

Im Zusammenhang mit der Notwendigkeit des adäquaten Umgangs mit M/O und der Palliativmedizin im Allgemeinen wurde mehrfach der Wunsch nach Lehre, Weiterbildung und **Wissen**sweitergabe an verschiedene **Adressaten** geäußert: *Medizinisches Personal (Ärzt:innen, Pflege, Psycholog:innen, etc.), Patient:innen, Angehörige* und die *Allgemeinbevölkerung*.

**Gewünschte Inhalte der Weiterbildung und Handlungsempfehlungen** beinhalteten insbesondere die notwendige Wissensvermittlung zur *Symptomkontrolle mit M/O*. Auch erwähnt wurden *Leitlinien zu COVID-19 und Palliativmedizin* sowie *Handlungsempfehlungen zu Opioiden im Sterbeprozess*.

Unsicherheit im Umgang mit M/O wurde auch mit unterschiedlichem *Konsens der Fachgesellschaften* assoziiert:

Mehr Konsens bei den Fachgesellschaften wäre hilfreich! Es gab in den letzten Jahren in der Fachpresse eine 180-Grad-Wendung von „wir verordnen zu wenig Opioide“ zu „wir verordnen viel zu viele Opioide“, ohne dass für mich nachvollziehbar wurde, warum – und ohne klare Maßstäbe, was „zu viel“ oder „zu wenig“ ist. (20/194, DGIM: Ärztin, Praxis, teilweise PM-Bereich))

Kategorie **(5) „Ergänzendes“** umfasste Anmerkungen, welche nicht in den vier anderen Hauptkategorien kodiert werden konnten. Hierzu zählten Anmerkungen zur Schilderung konkreter Aspekte zum** eigenen Eindruck** und **Themenvorschläge**.

[…] die Klagefreudigkeit schafft doch die vielen Probleme, weshalb Mediziner verunsichert sind. (131,/446, DGAI: Arzt, Krankenhaus, Praxis, Anästhesie/OP, Intensivmedizin, teilweise PM-Bereich)

## Diskussion

Dies ist eine qualitative Analyse der ergänzenden Freitextkommentare einer Online-Befragung von Mitgliedern mehrerer Fachgesellschaften zur Wahrnehmung des Umgangs mit Opioiden allgemein und bei COVID-19-Erkrankten [23, 24]. Die Auswertung belegte Unsicherheiten im Umgang mit Opioiden, die sich in den bereits publizierten quantitativen Daten zeigten: So nahmen drei von vier Ärzt:innen (Mitgliedschaft DGAI/BDA) den Umgang mit M/O bei der Betreuung COVID-19-Erkrankter nicht als „klar und geregelt“ für den Bereich *außerhalb* der Palliativmedizin (PM) wahr. Bedenkt man die teils heterogenen Empfehlungen unterschiedlicher Leitlinien zur Therapie der Dyspnoe während des Umfragezeitpunkts [16–18, 28], so wundert diese wahrgenommene Unsicherheit und Unklarheit nicht. Vor dem Hintergrund der aktuellen COVID-19-Pandemie, die das Personal durch steigenden Druck und Leid der Patient:innen belastet [26], erscheint ein sicherer Umgang mit Opioiden umso wichtiger.

Einerseits wird vor unsachgemäßer Verordnung von Opioiden und nichtmedizinischem Gebrauch im Rahmen der Opioidkrise in den USA gewarnt [2], und auch für Deutschland werden sich verändernde Handlungsempfehlungen beschrieben [22]. Aus bevölkerungsbasierten Studien ist sogar eine erhöhte Mortalität bei Patient:innen mit chronischen Nichttumorschmerzen unter der Einnahme starker Opioide bekannt, verglichen mit Personen ohne chronische Schmerzen [27]. Andererseits sehen internationale Autoren durch die aktuell restriktive Haltung eine Unterversorgung von Patient:innen mit Krebs- oder postoperativen Schmerzen [2].

Diese wechselnden Empfehlungen im Laufe der Jahre, die von anderen Autoren sogar als „Opioidpendel zwischen unkritischer Indikation und überkritischer Restriktion“ bezeichnet wurden [22], werden von einigen Teilnehmenden kritisiert. Es stellt sich die Frage, warum verschiedene Fachgesellschaften zu teils unterschiedlichem Konsens finden, wenn doch Empfehlungen zur Therapie standardisiert erfolgen und auf derselben verfügbaren Literatur basieren [3, 5]. Die zum Zeitpunkt der Umfrage aktuelle S3-Leitlinie „Empfehlungen zur stationären Therapie von Patienten mit COVID-19“ hingegen veranschaulicht erneut, dass bislang vorhandene allgemeine Empfehlungen zur Symptomkontrolle von Dyspnoe und Schmerz nicht selbstverständlich übernommen wurden: Worte wie „Opioide“ und „Benzodiazepine“ sowie konkrete Medikamentenangaben zur Symptomkontrolle fanden erst während der dritten Pandemiewelle Einzug in die revidierte Version dieser Leitlinie vom 17.05.2021, bei der in aktualisierter Form auch die Deutsche Gesellschaft für Palliativmedizin beteiligt war [16, 17].

Ein aktueller Artikel zum Schmerzmanagement in der Inneren Medizin [9] beleuchtet Schwierigkeiten bei einer bundesweiten Erhebung zu Struktur- und Prozessdaten, und beschreibt ein „häufig ausdrückliches Desinteresse am Thema Schmerzmanagement“. Die Autor:innen folgern auf der Basis vorhandener Literatur, dass „Defizite in der Versorgungsqualität im konservativen Bereich denen der operativen Bereiche entsprechen“ sowie Hinweise auf eine noch ausgeprägtere „Unter- und Fehlversorgung mit ausbleibender oder verzögerter Therapie von Schmerzen in den nicht-operativen Bereichen“. In ihrer eigenen Erhebung sehen sie Verbesserungspotenzial bei organisatorischen Rahmenbedingungen wie der Implementierung von Behandlungsstandards, schriftlichen Vereinbarungen und regelmäßiger Qualitätssicherung. Sie empfehlen, der Erfassung von „patient-reported outcomes“ zukünftig eine größere Bedeutung zukommen zu lassen.

Die Ergebnisse unserer Studie festigen den Eindruck von Wissens- und Erfahrungsdefiziten im Bereich des Umgangs mit Opioiden im ärztlichen und pflegerischen Bereich [23]. Die aktuelle qualitative Analyse veranschaulicht, dass die Wahrnehmungen zum Umgang mit Opioiden von einem selbstverständlichen Umgang bis hin zu Ängsten, wahrgenommenem Fehlgebrauch und großer Unsicherheit zu Regelungen bezüglich des Umgangs mit Opioiden reichen. Spezifische Lehre und Fortbildungsangebote könnten hier helfen, unbegründete Befürchtungen abzubauen oder zu relativieren.

Hingegen zeigen Anmerkungen wie „Atemdepression ist beim sinnvollen Umgang mit Opioiden eine therapeutische Stärke“ ein tieferes und fundiertes Verständnis im Gebrauch von Opioiden, denn die Gabe von Opioiden wirkt sich senkend auf die Atemfrequenz bei Tachypnoe aus [25]. Diese differenzierte Wahrnehmung sollte in zukünftigen Leitlinien entsprechend dargestellt werden, um Kolleg:innen mit wenig Erfahrung im Umgang mit Opioiden die Wirkung zu verdeutlichen und einer übertriebenen „Opiophobie“ entgegenzuwirken.

Die fachliche Unterstützung kann auch durch einen weiteren Ausbau der Palliativmedizin gefördert werden. So wurde schon zu Beginn der Pandemie der Palliativmedizin eine Schlüsselrolle in der Versorgung von Patient:innen zugeschrieben. Sie wurde als „essenzielle Antwort auf die COVID-19-Pandemie“ bezeichnet, da sie flexibel und bedarfsangepasst reagieren konnte [6, 19, 26]. Für die Kardinalsymptome Dyspnoe und Unruhe von COVID-19-Erkrankten in palliativer Situation wurden Opioide und Benzodiazepine mit gutem Erfolg bereits in niedrigen Dosierungen verabreicht [1, 13, 19].

Insbesondere einem palliativmedizinischen Konsildienst wird eine große Bedeutung zugemessen, was sich die Teilnehmenden auch in der quantitativen Analyse eindeutig gewünscht hatten [23]. Ein interdisziplinär tätiger Konsildienst kann sowohl direkt Patient:innen einschätzen und mitbehandeln als auch eine wertvolle Unterstützung für die Kolleg:innen anderer Fachrichtungen darstellen. Da Anästhesist:innen insbesondere interdisziplinär arbeiten und die Palliativmedizin oft mit der Anästhesie oder Inneren Medizin assoziiert ist, liegt ein weiterer, möglichst patienten- und bedarfsorientierter Ausbau dieser Strukturen nahe [2, 10, 14].

Ausbildung und Lehre insbesondere im Rahmen früher Integration von Palliativmedizin sind wichtig [8]. Einer frühen Integration von Palliativmedizin sowie ihrer übergeordneten, interdisziplinären Organisation wird von internationalen Autor:innen nicht nur eine Verbesserung im klinischen Outcome und z. B. Aspekten wie einer Vorbeugung von nichtmedizinischem Opioid-Verhalten zugeschrieben [2, 15, 21], sondern auch eine besondere Rolle in Forschung und Ausbildung von medizinischem Personal [2]. Die Etablierung einer solch übergeordneten Versorgungsstruktur in der Palliativmedizin könnte sogar eine organisatorische Resilienz fördern [2, 4, 11, 14] und lösungsorientiert viele hier von den Teilnehmenden genannte Probleme adressieren.

## Limitierung der Studie

Von einigen Teilnehmenden wurde trotz erläuternder Einführung im Fragebogen kritisiert, dass nicht immer klar war, um welche Situationen es sich in den gestellten Fragen handelte. So wurde geäußert, dass die Unterscheidung zwischen innerhalb und außerhalb der PM bzw. der Wahrnehmung des eigenen Fachbereichs versus des Bereichs anderer teils als unscharf wahrgenommen wurden. Der Bereich „außerhalb der PM“ bezieht sich auf sämtliche andere Fachbereiche und kann sehr heterogen verstanden worden sein. Zusammengefasst sollte die Bereichszuordnung als grobe Einschätzung eingeordnet werden. Ebenso wurde die Verwendung des Begriffs „Morphin“ stellvertretend für „Opioide“ kritisiert.

Einige Teilnehmende äußerten selbst, dass sie keine COVID-19-Erkrankten behandelt hätten, sodass sie zu Fragen mit COVID-19-Bezug nicht Stellung nehmen konnten.

## Fazit

Die Unsicherheiten im Umgang mit Opioiden sind vielfältig und sollten unbedingt durch mehr Lehre, einen weiteren Auf- und Ausbau von Palliativdiensten in Krankenhäusern sowie einheitliche, fachübergreifende Leitlinien mit standardisierten medikamentösen Empfehlungen zur Symptomkontrolle adressiert werden.
